# Biomonitoring of Lead, Cadmium, Total Mercury, and Methylmercury Levels in Maternal Blood and in Umbilical Cord Blood at Birth in South Korea

**DOI:** 10.3390/ijerph121013482

**Published:** 2015-10-26

**Authors:** Yu-Mi Kim, Jin-Young Chung, Hyun Sook An, Sung Yong Park, Byoung-Gwon Kim, Jong Woon Bae, Myoungseok Han, Yeon Jean Cho, Young-Seoub Hong

**Affiliations:** 1 Department of Preventive Medicine and Dong-A University Heavy Metal Exposure Environmental Health Center, Dong-A University College of Medicine, 49201 Busan, Korea; E-Mails: kimyumi@dau.ac.kr (Y.-M.K.); medikim@dau.ac.kr (B.-G.K.); 2 Dong-A University Heavy Metal Exposure Environmental Health Center, Dong-A University College of Medicine, 49201 Busan, Korea; E-Mail: jchung@dau.ac.kr; 3 Department of Obstetrics and Gynecology, Ilsin Christian Hospital, 48724 Busan, Korea; E-Mail: banana3592@hanmail.net; 4 New-born Obstetrics Clinic, 49340 Busan, Korea; E-Mail: dauhpsy@hanmail.net; 5 Department of Obstetrics and Gynecology, Dong-A University College of Medicine, 49201 Busan, Korea; E-Mails: jwbae@dau.ac.kr (J.W.B.); hmsobgy@dau.ac.kr (M.S.H.); jeanjane@dau.ac.kr (Y.J.C.)

**Keywords:** heavy metals, lead, cadmium, mercury, methylmercury, biomonitoring, pregnancy, umbilical cord

## Abstract

With rising concerns of heavy metal exposure in pregnancy and early childhood, this study was conducted to assess the relationship between the lead, cadmium, mercury, and methylmercury blood levels in pregnancy and neonatal period. The study population included 104 mothers and their children pairs who completed both baseline maternal blood sampling at the second trimester and umbilical cord blood sampling at birth. The geometric mean maternal blood levels of lead, cadmium, total mercury, and methylmercury at the second trimester were 1.02 ± 1.39 µg/dL, 0.61 ± 1.51 µg/L, 2.97 ± 1.45 µg/L, and 2.39 ± 1.45 µg/L, respectively, and in the newborns, these levels at birth were 0.71 ± 1.42 µg/dL, 0.01 ± 5.31 µg/L, 4.44 ± 1.49 µg/L, and 3.67 ± 1.51 µg/L, respectively. The mean ratios of lead, cadmium, total mercury, and methylmercury levels in the newborns to those in the mothers were 0.72, 0.04, 1.76, and 1.81, respectively. The levels of most heavy metals in pregnant women and infants were higher in this study than in studies from industrialized western countries. The placenta appears to protect fetuses from cadmium; however, total mercury and methylmercury were able to cross the placenta and accumulate in fetuses.

## 1. Introduction

Lead, cadmium, and mercury are naturally distributed heavy metals and have been commonly used for thousands of years. Their health hazards after high dose exposure in occupational workplaces or accidental leakages are well known. Recently, the adverse health effects of chronic and/or low dose exposure of these heavy metals as well as the health effects in sensitive populations have been highlighted [1,2,3]. According to 2013 Priority List of Hazardous Substances presented by the Agency for Toxic Substances and Disease Registry, Pb (2nd), Cd (7th), and Hg (3rd) were highly ranked heavy metals.

The adverse effects of heavy metal exposure in the prenatal and early childhood periods are of increasing concern in terms of high exposure on a body weight basis, immature metabolic pathways, delicate developmental processes, and life course effects [4]. Lead and mercury have been shown to be easily transferred through the placental barrier and blood brain barrier [5,6]. Additionally, adverse outcomes in pregnancy, such as fetal loss [7] and retarded fetal growth [8], have been shown to be associated with prenatal lead exposure. Prenatal exposure to lead and mercury has been related to congenital anomalies [9,10] and impaired neurodevelopment [11,12]. Although the placenta has been reported to be a partial barrier for cadmium [5,6], some studies have presented the harmful health effects of prenatal cadmium exposure [13,14].

Considering no safe maternal exposure levels of heavy metals have been presented and the possibility of placental transfer, reliable monitoring of blood levels of heavy metals in pregnancy and early childhood has been suggested [15].

Importance of biomonitoring for both mother and child as the environmental exposure sensitive population, requires more updated data and studies. However, to our knowledge, only a few studies on heavy metal levels in Korean mother-child pairs, including umbilical cord blood sampling, have been published [16,17,18].

The purpose of the present study was to summarize the lead, cadmium, mercury, and methylmercury blood levels in pregnancy with maternal blood sampling and in the neonatal period with umbilical cord blood sampling at delivery in South Korea.

## 2. Experimental Section

### 2.1. Study Participants

**Figure 1:**
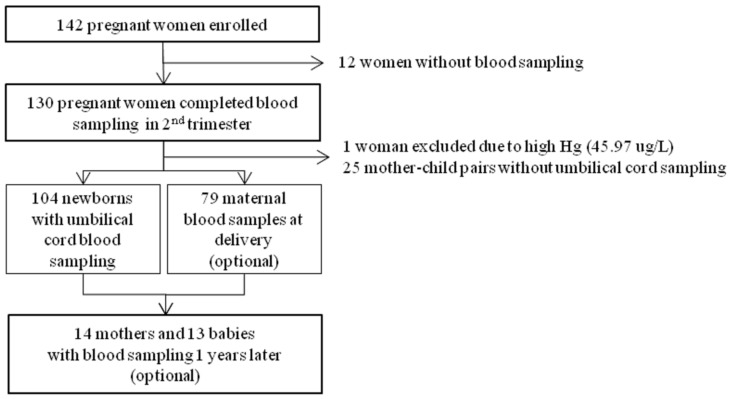
Flowchart of the study population.

A total of 142 pregnant women from three obstetric clinics in Busan Metropolitan City (located at the southwest coast of the Korean peninsula with a population of 3,500,000) voluntarily participated in the present study in 2013. Of the 142 pregnant women, 130 underwent baseline maternal blood sampling at the second trimester. Additionally, umbilical venous cord blood sampling at delivery was performed in 105 women. One woman who had a very high blood level of mercury (45.97 µg/L) was recommended to undergo further clinical evaluation and was excluded from the study. Therefore, 104 mother-child pairs were finally included in the analysis. In a previous pilot study, most of the pregnant women refused additional blood collection because of the relatively large blood volume required for routine clinical checkups at delivery. Thus, in the present study, maternal blood sampling was optional. Blood samples at delivery were collected from 79 women. The study participants were requested to undergo a re-examination after 1 year, and 14 mother-child pairs accepted the request (Figure 1). The Institutional Review Board of Dong-A University Hospital approved the study.

### 2.2. Questionnaire

A simple questionnaire was developed to gather information on mother’s age, gestational weeks at recruitment and delivery, delivery method, and infant’s characteristics including sex, birth order, and hospitalization after birth. The questionnaire was filled out twice by two well-trained personnel, who administered the questionnaire in a face-to-face manner at the second trimester for basic information. After delivery, the variables including the delivery method and baby’s information were collected according to the hospital records.

### 2.3. Heavy Metals Analysis

Whole venous blood was directly collected using 3 ml vacuum blood collection tubes (Vacutainer^®^, Beckton & Dickton, Franklin Lakes, NJ, USA) treated with EDTA to prevent coagulation. Immediately after collection, the blood samples were stored at −20 °C until heavy metal analysis.

Lead and cadmium levels were determined using an inductively coupled plasma-mass spectrometer (ICP-MS) (Agilent Technologies 7700 series; Agilent Technologies, Santa Clara, CA, USA) equipped with a low flow sample introduction system and high matrix introduction kit (Agilent Technologies). The details of the operating condition of the ICP-MS are presented in Table 1. The detection limits for lead and cadmium using this system were 0.079 µg/dL and 0.002 µg/L, respectively.

**Table 1:** The operating conditions of the ICP-MS for lead and cadmium analyses.

Parameters	Conditions
RF Power (kW)	1.55
Carrier gas flow rate (L/min)	1.15
Sample depth (mm)	8.0
Sampler and skimemer cones	Nickel
Spray chamber temperature (°C)	2
Nebulizer type	Concentric nebulizer
Sample uptake rate (RPS)	0.5
He flow rate (mL/min)	4.5
Analytical masses	^111^Cd, ^208^Pb

Abbreviations: ICP-MS, inductively coupled plasma-mass spectrometer; RF, radio-frequency; RPS, resolution per second.

Total blood mercury levels were determined with the gold amalgamation method using an automatic mercury analyzer (SP-3DS; Nippon Instrument Corp., Tokyo, Japan). Methylmercury levels were determined with cold vapor atomic fluorescence spectrophotometry using an automated methylmercury analytical system (MERX; Brooks Rand Co., Seattle, WA, USA). Details of the mercury and methylmercury analyses have been presented by Kim *et al.* [19].

### 2.4. Statistical Analysis

The heavy metal levels were transformed by a natural logarithm because of their right-skewed distributions. The geometrical means and variances have been presented as descriptive statistics for each period. The longitudinal measurements and changes in heavy metal levels were estimated using linear mixed models, which consider correlations among measurements made on the same subject. Linear correlations between maternal blood levels of heavy metals and umbilical cord blood levels of heavy metals at birth were explored using scatter plots, and the strengths of linearity were estimated using Spearman’s correlation coefficients. A *p* value < 0.05 was considered statistically significant.

## 3. Results

### 3.1. General Characteristics of the Study Participants

At recruitment, the study participants were at 13 to 28 gestational weeks and their mean age was 31.8 ± 4.0 years (range, 22–46 years). The sex ratio of the infants (the number of girls divided by the number of boys) was 0.58 (Table 2).

**Table 2:** General characteristics of the 104 mother-child pairs.

Category	Characteristics	Mean	±Std	Min	Max
**Maternal characteristics**					
	Age (year)	31.8	±4.0	22.0	46.0
	Gestational weeks at recruitment (week)	20.7	±4.3	13.0	28.0
	Gestational weeks at delivery (week)	38.8	±1.2	34.6	41.1
**Infant’s characteristics**		n	(%)		
	**Sex**				
	boy	66	(63.5)		
	girl	38	(36.5)		
	**Delivery methods**				
	vaginal	65	(62.5)		
	C/S	39	(37.5)		
	**Birth order**				
	first	53	(51.0)		
	over second	51	(49.0)		
	**Hospitalization after birth**				
	no	78	(75.0)		
	yes	26	(25.0)		

Abbreviations: Std, Standard deviation; Min, minimum; Max, maximum; N, number; C/S, cesarean section.

### 3.2. Maternal Blood Levels of Heavy Metals at Different Sampling Times

The geometric mean maternal blood levels of heavy metals at the second trimester, delivery, and 1 year after delivery are presented in Table 3.

The geometric mean lead and cadmium levels did not differ among the three sampling times. The geometric mean total mercury levels at the second trimester, delivery, and 1 year after delivery were 2.97 (±1.45) µg/L, 2.66 (±1.40) µg/L, and 3.63 (±1.48) µg/L, respectively, and the levels were significantly different among the three sampling times (*p* = 0.0079). On *post hoc* analyses, the mean total mercury levels were significantly higher at 1 year after delivery than at delivery (*p* = 0.0045) and were significantly higher at the second trimester than at delivery (*p* = 0.0391). The geometric mean methylmercury levels at the second trimester, delivery, and 1 year after delivery were 2.39 (±1.45) µg/L, 2.16 (±1.42) µg/L, and 3.25 (±1.49) µg/L, respectively, and the levels were significantly different among the three sampling times (*p* = 0.0010). On *post hoc* analyses, the mean methylmercury levels at 1 year after delivery were significantly higher than at the second trimester (*p* = 0.0044) and at delivery (*p* = 0.0003).

**Table 3:** Maternal blood levels of heavy metals at the second trimester, delivery, and 1 year after delivery.

Heavy Metals	Periods	N	GM	G. Std	Min	P10	P25	P50	P75	P90	Max	*p* Value *
Lead	at 2nd trimester	104	1.02	±1.39	0.50	0.65	0.81	1.03	1.26	1.58	2.20	0.7703
(µg/dL)	at delivery	79	1.03	±1.34	0.51	0.67	0.85	1.02	1.27	1.51	1.82	
	after 1 year	13	1.08	±1.34	0.66	0.81	0.89	1.02	1.48	1.58	1.62	
Cadmium	at 2nd trimester	104	0.61	±1.51	0.26	0.38	0.44	0.60	0.81	1.01	2.01	0.6437
(µg/L)	at delivery	79	0.61	±1.58	0.24	0.33	0.45	0.59	0.84	1.09	2.80	
	after 1 year	13	0.54	±1.52	0.23	0.35	0.39	0.61	0.79	0.84	0.86	
Total mercury	at 2nd trimester	104	2.97	±1.45	1.34	1.92	2.34	2.89	3.71	5.05	8.41	0.0079
(µg/L)	at delivery	79	2.66	±1.40	1.36	1.75	2.14	2.66	3.13	4.26	8.50	
	after 1 year	13	3.63	±1.48	2.23	2.36	2.56	3.42	4.90	5.87	7.77	
Methylmercury	at 2nd trimester	104	2.39	±1.45	1.02	1.54	1.86	2.25	2.89	4.18	6.96	0.0010
(µg/L)	at delivery	79	2.16	±1.42	1.02	1.40	1.72	2.18	2.59	3.32	6.82	
	after 1 year	13	3.25	±1.49	1.88	1.95	2.41	2.86	4.26	5.26	7.03	

* *p* values for differences among periods using a mixed model; Abbreviations: N, number; GM, geometric mean; G. Std, geometric standard deviation; Min, minimum; Max, maximum; P, percentile.

### 3.3. Blood Levels of Heavy Metals in the Children at Different Sampling Times

The geometric mean blood levels of heavy metals in the children at birth and 1 year of age are presented in Table 4.

The geometric mean cadmium level was significantly lower at birth than at 1 year of age (*p* = 0.0222). The mean total mercury and methylmercury levels at birth were significantly higher than at 1 year of age (both *p* < 0.0001).

**Table 4:** Blood levels of heavy metals in the children at birth and 1 year of age.

Heavy Metals	Periods	N	GM	G. Std	Min	P10	P25	P50	P75	P90	Max	*p* Value *
Lead	at birth	104	0.71	±1.42	0.47	0.57	0.73	0.88	1.05	0.20	1.58	0.1161
(µg/dL)	at 1 year old	14	0.85	±1.73	0.46	0.55	0.84	1.49	1.90	0.39	2.14	
Cadmium	at birth	104	0.01	±5.31	0.00	0.00	0.02	0.04	0.06	0.00	0.22	0.0222
(µg/L)	at 1 year old	14	0.03	±3.35	0.00	0.02	0.04	0.08	0.13	0.00	0.18	
Total mercury	at birth	104	4.44	±1.49	2.61	3.32	4.35	5.58	7.24	2.08	12.06	<0.0001
(µg/L)	at 1 year old	14	1.51	±1.52	0.90	1.22	1.45	1.99	2.53	0.64	3.49	
Methylmercury	at birth	104	3.67	±1.51	2.25	2.59	3.56	4.57	5.87	1.79	11.25	<0.0001
(µg/L)	at 1 year old	14	1.48	±1.48	0.85	1.28	1.45	2.03	2.16	0.71	3.25	

* *p* values for differences among periods using a mixed model; Abbreviations: N, number; GM, geometric mean; G. Std, geometric standard deviation; Min, minimum; Max, maximum; P, percentile.

### 3.4. Ratios of Heavy Metal Levels in the Newborns to those in the Mothers

For the assessment of heavy metal transfer from mothers to their fetuses, we calculated the ratios of heavy metal levels in the newborns to those in the mothers (Table 5).

Approximately 72%–76% of maternal lead was estimated to have been transferred to newborns. The mean ratio of cadmium levels in the newborns to those in the mothers was very low, except for outliers. The mean ratio of total mercury levels in the newborns at birth to those in the mothers at the second trimester was 1.56, and the mean ratio of methylmercury levels in the newborns at birth to those in the mothers at the second trimester was 1.62. In the 79 mother-child pairs assessed at birth, the mean ratios of total mercury and methylmercury levels were 1.76 and 1.81, respectively.

**Table 5:** Ratios of heavy metal levels in the newborns to those in the mothers.

Heavy Metals	N	Mean	Std	Min	P25	Median	P75	Max
Lead	104 pairs *	0.76	±0.31	0.19	0.56	0.72	0.89	2.52
	79 pairs **	0.72	±0.25	0.33	0.55	0.66	0.87	1.80
Cadmium	104 pairs	0.04	±0.05	0.00	0.01	0.03	0.05	0.42
	79 pairs	0.04	±0.05	0.00	0.01	0.02	0.06	0.37
Total mercury	104 pairs	1.56	±0.49	0.64	1.26	1.49	1.77	4.01
	79 pairs	1.76	±0.43	1.09	1.49	1.68	1.90	4.26
Methylmercury	104 pairs	1.62	±0.56	0.69	1.22	1.57	1.90	4.43
	79 pairs	1.81	±0.52	1.25	1.45	1.73	1.95	4.89

* mother at second trimester and newborn pairs, ** mother at delivery and newborn pairs; Abbreviation: N, number; Std, standard deviation, Min, minimum, Max, maximum; P, percentile.

### 3.5. Correlation between Maternal Blood Levels of Heavy Metals and Newborn Blood Levels of Heavy Metals at Birth

The Spearman’s correlation coefficients between mothers at the second trimester and newborns from 104 pairs were 0.40 (95% CI: 0.23–0.55) for lead, 0.43 (95% CI: 0.26–0.57) for cadmium, 0.70 (95% CI: 0.59–0.79) for total mercury, and 0.65 (95% CI: 0.53–0.75) for methylmercury.

**Figure 2:**
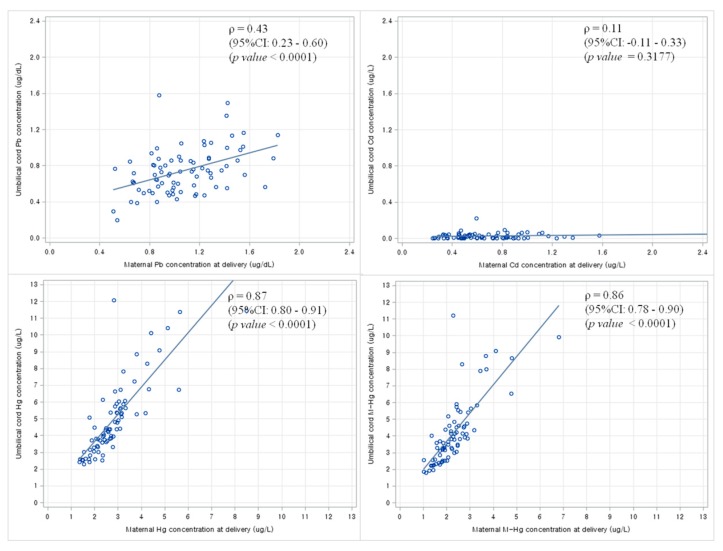
Scatter plots and Spearman’s correlation analysis between maternal blood levels of heavy metals at the delivery and newborn blood levels of heavy metals at birth from 79 pairs. Abbreviations: ρ, Spearman’s correlation coefficient; CI, confidence interval.

Using scatter plots and Spearman’s correlation analysis, linear trends were noted between maternal blood levels of heavy metals at delivery and newborn blood levels of heavy metals at birth from 79 mother-child pairs (Figure 2).

## 4. Discussion

To our knowledge, only three studies on heavy metal levels in Korean mother-child pairs, including umbilical cord blood sampling, have been reported. These Korean studies have reported results similar to our results for lead [16] and mercury [17,18]. The levels of lead and mercury in Korean mother-child pairs did not appear to rise over the past 3–5 years based on these previous reports. The descriptive statistics from this study would update current heavy metals exposure conditions in pregnant women and children who have been calling for public and political attention as the environmental hazards sensitive and/or vulnerable population in Korea.

The blood levels of lead and mercury in pregnant women in the U.S. have been reported to be 0.64 µg/dL and 0.69 µg/L, respectively, using national representative data, which are lower than our values [20]. Additionally, the geometrical mean levels of lead, total mercury and methylmercury were higher in our study than in a study with Canadian Caucasians, which reported that the blood level of lead, cadmium, total mercury and methylmercury were 2.06 µg/dL, 0.43 µg/L, 0.87 µg/L and 0.69 µg/L, respectively [21]. Especially, the levels of mercury and methylmercury from this study were the relatively higher as compared with the values from the U.S. and Canada. The main source of human mercury exposure is known to be mercury contaminated fish and shellfish. The higher levels of mercury and methylmercury in our study than in the previous studies from Western countries might be explained by the presence of lifestyle risk factors for mercury exposure in Korea, such as high seafood consumption [22]. As an example with high seafood consumption country like Korea, Sakamoto *et al.* has reported that the mean maternal RBCs level of lead and mercury were 26.4 ng/g and 9.41 ng/g from Japanese women [23]. Additionally, high levels of mercury and methylmercury in pregnant women and children were also observed in studies from the Mediterranean [24] and Arctic region [25].

In mothers, the levels of mercury and methylmercury were found to be significantly higher 1 year after delivery than at the second trimester. However, in children, these levels were found to be lower at 1 year of age than at birth. The ratios of total mercury and methylmercury levels in the newborns to those in the mothers were 1.76 and 1.81, respectively. There was possibility that the pregnant women restricted the consumptions of fish and the baby might have a little chance to be exposed to mercury contaminated fish food. A meta-analysis with 10 studies in countries with significant fish consumptions reported that levels of methylmercury were approximately 1.7 times higher in newborns than in mothers [26]. Fetal accumulation of methylmercury has been known to result from active transfer with a neutral amino acid carrier [27]. These characteristics imply the importance of monitoring and control in mercury and methylmercury of pregnancy and infants considering the well-known adverse health effects.

Besides the correlation coefficient between mothers and newborns for cadmium indicated weak linear trend, the ratio of cadmium levels in the newborns to those in the mothers was very low. Metallothionein was suggested for the prevention of cadmium transfer from mothers to fetuses [28]. Despite the effectiveness of the placental barrier for preventing cadmium transfer, concerns about adverse health outcomes after prenatal cadmium exposure have been increasing. Maternal exposure to cadmium was related with early delivery and a lower birth weight [13]. A study has suggested the hypothesis between cadmium exposure and adverse effect of fetal development [14].

The ratio of lead levels in the newborns at birth to those in the mothers at delivery was 0.72 (interquartile range, 0.56–0.89). Partial transfer of lead from mothers to newborns though the placental barrier was noted in this study. As the degree of lead transfer may be affected by maternal conditions such as blood pressure, hemoglobin level, and alcohol use [29], the influencing factors should be evaluated in further studies.

The present study had several limitations. First, the sample size was small. The small number of participants restricted the statistical analyses and decreased the statistical power. The number of newborns in Busan Metropolitan City in 2013 was reported to be 25,800 [30]; however, only 104 mother-child pairs were included in the present study. The follow-up rate at 1 year after birth was also low. Considering that the main purpose of this study was to determine the levels of heavy metals in maternal blood during pregnancy and in umbilical cord blood at birth, the 1 year follow-up data may be considered as supplementary information. Second, convenient sampling and the small number of participants in this study might reduce the representativeness of the findings. Further studies involving biomonitoring with a larger and more representative population are needed. Third, the mothers who voluntarily participated in the second and third samplings after the baseline sampling may have had a healthy lifestyle and been more interested in perinatal health care. This might result in underestimation of the findings.

The present study has some advantages. This prospective study updated the data on heavy metals levels from early pregnancy to 1 year after delivery. The levels of heavy metals (lead, cadmium, total mercury, and methylmercury) were identified for 104 mother-child pairs in a coastal industrialized metropolitan city in Korea. Using umbilical cord blood, the role of the placenta in the transfer of heavy metals was shown. The ratio of lead levels in the newborns to those in the mothers was less than 1.0, showing the role of the placenta in the prevention of lead transfer. Moreover, accumulation of maternal total mercury and methylmercury in the fetus was shown.

Although the sample size was small, which could restrict the representativeness of the findings and statistical power, the data obtained may help in the development of strategies for the reduction of environmental heavy metal exposure in pregnant women and children. A long-term large scale study involving biomonitoring is necessary to provide further reliable data on heavy metal exposure, which will help prevent heavy metals exposure in pregnant women and children and protect their health.

## 5. Conclusions

This study summarized the lead, cadmium, mercury, and methylmercury blood levels in pregnancy and the neonatal period in a rapidly economically developed country where people usually eat fish. The levels of most heavy metals in pregnant women and infants were higher in this study than in studies from industrialized western countries, however, the levels were similar to those in studies from the coastal area. The placenta appears to protect fetuses from cadmium; however, total mercury and methylmercury are able to cross the placenta and accumulate in fetuses.
